# Leishmaniasis in Humans and Animals: A One Health Approach for Surveillance, Prevention and Control in a Changing World

**DOI:** 10.3390/tropicalmed9110258

**Published:** 2024-10-28

**Authors:** Claudia Cosma, Carla Maia, Nushrat Khan, Maria Infantino, Marco Del Riccio

**Affiliations:** 1 Department of Health Sciences, University of Florence, 50134 Florence, Italy; 2 Global Health and Tropical Medicine (GHTM), Associate Laboratory in Translation and Innovation Towards Global Health (LA-REAL), Instituto de Higiene e Medicina Tropical (IHMT), Universidade NOVA de Lisboa (UNL), Rua da Junqueira 100, 1349-008 Lisboa, Portugal; 3 Department of Primary Care and Public Health, School of Public Health, Faculty of Medicine, Imperial College, 90 Wood Ln, London W12 0BZ, UK; 4 Immunology and Allergology Laboratory Unit, S. Giovanni di Dio Hospital, Azienda USL-Toscana Centro, 50012 Florence, Italy

**Keywords:** neglected tropical diseases, vector-borne diseases, sandflies, climate change, urbanisation, migration, globalisation

## Abstract

Leishmaniasis is classified as a neglected tropical disease (NTD), caused by protozoan parasites of the genus *Leishmania*, which are transmitted to humans and other animals through the bite of infected female phlebotomine sandflies. There are three forms of the disease: cutaneous leishmaniasis (CL) manifested by ulcers and scars; systemic or visceral leishmaniasis (VL), which can lead to life-threatening complications if left untreated; and mucocutaneous leishmaniasis (MCL), which can destroy the mucous membranes of the nose, mouth and throat. Human leishmaniasis is endemic in many countries across Africa, Asia, Southern Europe, the Middle East, and Central and South America. The interconnection of environmental, animal and human health underlies the spread of the *Leishmania* parasite. Environmental disruptions, such as climate change, deforestation or urbanisation, but also globalisation and migration, significantly affect the distribution and abundance of sand fly vectors and reservoir hosts. Climate change alters the breeding patterns of sandflies and expands their geographic range; deforestation and misuse of large areas disrupt ecosystems, leading to increased human-vector contact; and urbanisation increases the potential for contact between parties, particularly in densely populated areas. Migration of humans and animals, either through natural migration or, for example, the pet trade and breeding, can facilitate the spread of *Leishmania* parasites. In addition, socio-economic factors, including poverty and lack of access to healthcare, increase the burden of leishmaniasis in vulnerable populations. Due to this multitude of reasons, the geographic distribution of sandflies has expanded to higher latitudes and altitudes in recent years, with a consequent increase in disease burden. Indeed, despite ongoing challenges in the surveillance systems, data from the last available year have shown an increase in many cases in both humans and dogs. This perspective explores the interconnected factors influencing the spread of leishmaniasis worldwide and the epidemiology of the disease. In addition, it illustrates the importance of integrated strategies in a One Health approach: surveillance, prevention and control of vectors, animals and humans.

## 1. Leishmaniasis in a Changing World

Leishmaniasis is one of the 20 neglected tropical diseases (NTDs) [1], which together affect more than one billion people worldwide [2]. This NTD is endemic in 99 countries [3] and occurs in multiple forms. It is typically associated with poverty and often exacerbates it, as it frequently prevents people from working, socialising or attending school, thus perpetuating a vicious cycle of marginalisation. Tropical and subtropical climates favour its spread; however, the epidemiology of leishmaniasis has changed significantly in recent years. Climate emergency and global warming, along with globalisation, migration and increased human and animal mobility, are therefore intensifying the spread of the disease. Additionally, wars alter local ecosystems and force populations to move, further contributing to and increasing the risk gradient [4]. In the case of leishmaniasis, there is a further complicating factor. There are about 70 species of animals, including humans and dogs, that are the source of parasites [1,5,6]. The large variety of reservoir hosts further complicates the identification and controlling of transmission.

In light of this, in a changing world, with an environment undergoing profound alterations and the consequent redefinition of human-animal relationships, the landscape of leishmaniasis is changing, and with it, the burden of disease. The aim of this perspective is to raise awareness of the current epidemiological state of knowledge, advocating guidance on the re-evaluation of surveillance, prevention and control strategies in the only way that can be successful in combating the disease: an integrated One Health approach.

## 2. An Overview of the Clinical, Epidemiological and Economic Burden of *Leishmaniasis*

**Figure 1:**
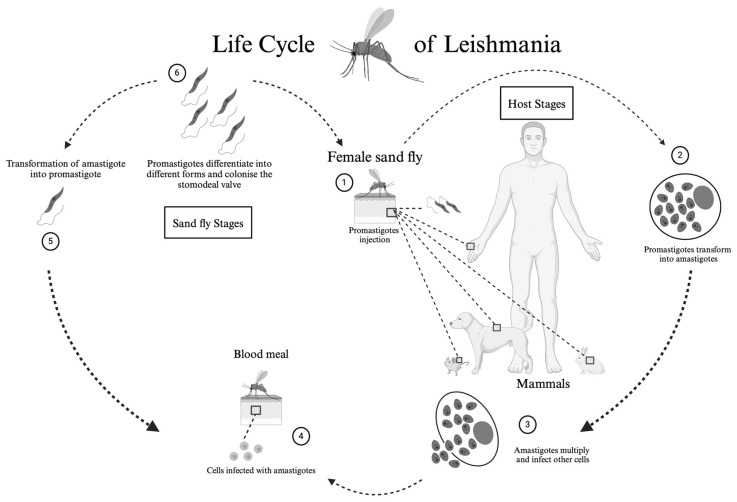
**Life cycle of the *Leishmania* protozoan parasite.** (**1**) The cycle begins when the sand fly inoculates metacyclic (infective) promastigotes into the vertebrate host during a blood meal, along with the fly’s saliva, midgut microbiota, and extracellular vesicles of the parasite. (**2**) The promastigotes are phagocytosed by macrophages and other mononuclear cells, transforming into amastigotes. (**3**) The amastigotes divide and infect other cells. (**4**) The sand fly, during a subsequent blood meal, ingests the infected cells. (**5**) In the midgut of the sand fly, the amastigotes transform into promastigotes. (**6**) The promastigotes differentiate into metacyclic forms and colonise the stomodeal valve. Created in BioRender. Cosma, C. (2024). https://biorender.com/l08n361.

Leishmaniasis is a disease caused by protozoan parasites of the genus *Leishmania*, transmitted through the bite of an infected female phlebotomine sand fly (Figure 1). The genera of sandflies most frequently involved in transmission are *Phlebotomus* in the Old World and *Lutzomyia* in the New World.

Leishmaniasis occurs in three main clinical forms: cutaneous leishmaniasis (CL), the most common form that manifests through ulcerated lesions of the skin that can leave permanent scars [7]; mucocutaneous leishmaniasis (MCL), which affects the mucous membranes of the nose, mouth and throat; and visceral leishmaniasis (VL), which is the most severe form [8]. Post-kala-azar dermal leishmaniasis (PKDL) is a complication of VL that may manifest as a macular, maculopapular and nodular rash after treatment of VL [9]. There are more than 20 pathogenic species, with different geographical distributions. The most relevant include *L. infantum*, which is responsible both for VL, also known as kala-azar, and cutaneous leishmaniasis (CL); *L. donovani* for the VL and PKDL; *L. major*, *L. tropica* and *L. mexicana*, which cause CL, with the infection caused by *L. tropica* being referred to as “oriental button”; and *L. braziliensis*, which causes CL and MCL [10,11].

The disease is characterised by a complex pathogenesis, which can vary from an overactive to an ineffective immune response and can generate a wide variety of specific symptoms that often confuse the diagnostic picture and mimic other inflammatory or infectious diseases [12,13]. The impact of leishmaniasis on human health can be very serious depending on the form of the disease. VL causes systemic effects such as prolonged fever, weight loss, splenomegaly, hepatomegaly and pancytopenia. If left untreated, VL can be fatal due to complications such as haemorrhage, secondary infections or liver dysfunction. CL, while generally not life-threatening, can cause disfiguring skin ulcers that can lead to scarring, with psychological and social consequences. MCL is commonly more severe than CL, resulting in significant morbidity if left untreated [10].

Similar to humans, the clinical picture of animal leishmaniasis, particularly in dogs, can vary widely, ranging from an asymptomatic course to severe systemic disease. In dogs with clinical leishmaniasis, clinical signs such as progressive weight loss, diffuse or localised skin lesions, especially on the snout, ears, and limbs, generalised lymphadenopathy, and, in the most severe cases, chronic renal failure, which is a major cause of death in infected individuals, are frequently observed. Other clinical signs include epistaxis, muscle atrophy, and ocular lesions such as uveitis [14,15].

Along with the considerable health consequences, leishmaniasis deserves attention for its significant economic burden on both the human health system and the animal sector. Healthcare costs for treatment (often substantial) and loss of productivity due to disability in recovered patients have a particularly heavy impact in low- and middle-income countries (LMICs), where the disease is more widespread and there is limited access to healthcare services [16]. For this reason, the social burden cannot be underestimated either: on society as a whole, the disease leaves scars not only on the body, but also by reducing educational and socio-economic outcomes, aggravating poverty and economic fragility. Worsening the health conditions of sufferers and even of those cured of leishmaniasis are behaviours of social isolation, stigma, blame, and discrimination [17]. Compared to all NTDs, leishmaniasis contributes to about 4% of Disability-Adjusted Life Years (DALYs) (VL alone carries a global annual burden of up to 1.6 million DALYs) and 5.5% of all deaths related to these diseases. These estimates are likely significantly underestimated due to underreporting of cases and a lack of adequate resources for diagnosis, treatment, and prevention [18,19,20]. The primary economic burden of human leishmaniasis concerns VL, which requires hospitalization and prolonged pharmacological treatments with devastating consequences for the economy. In fact, the treatment of VL entails high direct medical expenses, coupled with indirect costs related to income loss during the illness. This scenario worsens the economic situation of already vulnerable families, who are often forced to resort to non-conventional financing and incur debt through high-interest rates to cover medical expenses [21]. The lack of easily accessible healthcare infrastructure and the economic pressure arising from medical expenses cause further impoverishment, reducing the prospects for economic recovery both for families and for the community as a whole. In countries like India, Sudan, and Brazil, where leishmaniasis is particularly widespread, the economic impact of the disease is even more evident. The distance of public healthcare facilities from villages where most infected people live pushes inhabitants to seek care at local private healthcare facilities, which are more expensive but often inadequate. For example, in India, the average cost to treat VL can amount to 17.5% of family income, and in the worst cases, families may end up spending up to 72% of their total resources to cope with the disease. This situation obviously fuels a vicious cycle of poverty, making it difficult to overcome the economic and health crisis [21].

Managing the disease is not more advantageous or cost-effective in the animal sector, particularly regarding dogs. Veterinary care for diseased dogs is expensive and often requires long-term management of chronic diseases. This represents an additional economic burden for families, particularly in rural areas where the disease is endemic.

### 2.1. Epidemiology of Leishmaniasis in Humans

Recent estimates indicate that between 700,000 and one million new cases of leishmaniasis occur each year worldwide [1]. In recent years, leishmaniasis has shown an increasing geographical spread, with supposedly autochthonous human cases reported in previously non-endemic areas, including Western Europe and North America. Factors amplifying this spread include the climate emergency and global warming, along with migration and a constant increase in international travel [1,22].

In East Africa, where 73% of cases of VL are concentrated, half of the global infections occur among children under 15 years of age. About 95% of CL is concentrated in the Americas, the Mediterranean basin, the Middle East and Central Asia where climatic, socioeconomic factors and internal migrations favour the spread of the disease [14]. MCL cases are mainly concentrated in Bolivia, Brazil, and Peru, which are considered endemic areas [23].

**Figure 2:**
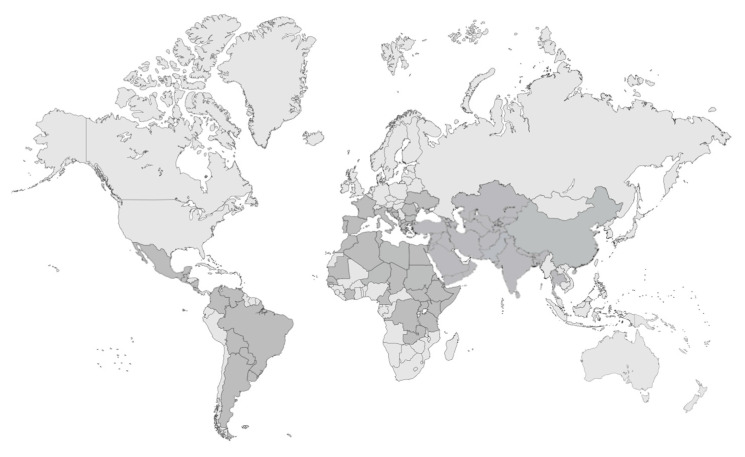
**Visceral leishmaniasis: status of endemicity.** Map showing countries endemic for visceral leishmaniasis according to WHO data, highlighting regions where the disease is present and actively transmitted [39]. Data updated to 2022. Created in BioRender. Cosma, C. (2024). https://biorender.com/o09z672.

**Figure 3:**
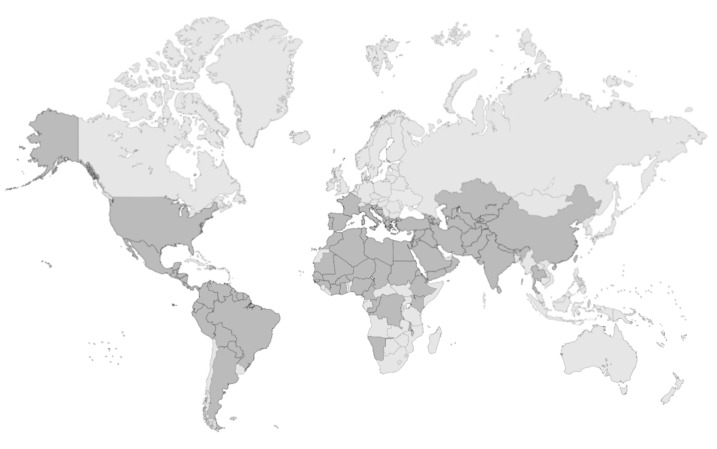
**Cutaneous leishmaniasis: status of endemicity.** Map showing countries endemic for cutaneous leishmaniasis according to WHO data, highlighting regions where the disease is present and actively transmitted [40]. Data updated to 2022 [40]. Created in BioRender. Cosma, C. (2024). https://biorender.com/g93i062.

The varying geographical distribution of leishmaniasis (Figure 2 and Figure 3) around the world is influenced by various factors, including the species of parasite involved, the presence of specific vectors and environmental and socio-economic conditions. For example, in Europe the most widespread species is *L. infantum* [24], also present in China, Latin America, the Middle East and several countries of North Africa, although in Latin America, the predominant species are *L. braziliensis* and *L. mexicana* [10,25]; in Asia, the most widespread species is *L. donovani*, while in Africa, *L. donovani*, *L. major* and *L. tropica* species are prevalent [10,26]. Humans can also act as reservoir hosts of the parasite, resulting in a form of anthroponotic VL from *L. donovani* confined almost to East Africa and the Indian subcontinent [27].

In Europe, leishmaniasis caused by *L. infantum* expanded significantly from 2005 to 2020, spreading to areas such as northern Italy and some regions of France [24], particularly due to increased climate suitability [28]. Considering the current factors driving the disease’s expansion, the civil war in Syria serves as a prominent example. The collapse of public health systems and mass migrations due to war have facilitated the spread of *L. tropica*, a species for which humans are the primary reservoir hosts, to countries such as Turkey, Lebanon, and Jordan [29,30]. Similarly, in Latin America, deforestation and urbanisation have exposed new populations to the risk of infection, increasing the spread of the disease in rural and urban areas. Countries such as Colombia, Brazil, Costa Rica and Peru, which are among the ten with the highest estimated number of CL cases, contribute 70–75% of the global incidence of this disease. Among the most involved species are *L. braziliensis* for CL, and *L. infantum* (also known as *L. chagasi* in these regions) for VL [31,32].

Urban expansion itself and the alteration of natural habitats have contributed to the creation of ideal conditions for the spread of the disease: a significant example is the epidemic of CL and VL that occurred in Fuenlabrada, Madrid, between 2009 and 2012. This outbreak, the largest ever recorded in Western Europe, involved over 400 people [33]. The outbreak was associated with the proliferation of hares, a sylvatic peridomestic reservoir for *L. infantum*, which, through sandflies, then spread to humans [34]. This episode reminds us that leishmaniasis can no longer be considered solely a rural disease, as it is increasingly becoming an urban problem as well.

In addition, the underreporting and underestimation of cases remain an open issue, well highlighted by studies suggesting that the actual number of infections may be a multiple of what is officially reported by health authorities [31]. For example, a study conducted in Spain between 2016 and 2017 confirmed a predictable gap between reported parasitic infections and their actual estimated number [35]. This underreporting issue is present in many other countries, e.g., in Portugal and Italy [24], despite the frequent mandatory notification for physicians and veterinarians. Indeed, by testing blood donors, there is a growing number of studies reporting very high prevalences of antibodies (approximately 16% to 41%) against the *Leishmania* parasite in populations living in endemic areas, underlining the possibility of underreporting of cases [36,37,38].

### 2.2. Epidemiology of Leishmaniasis in Animals

Leishmaniasis can affect many different animals. Dogs are the primary reservoirs for leishmaniasis specifically caused by *L. infantum* in various regions of the Old and New Worlds [41], and they themselves are also affected. Many wild animals also play the role of hosts. Small rodents are reservoirs for *L. major* in the Old World and *L. braziliensis* in the New World, while other indigenous animals, such as marsupials, sloths, and monkeys, are the main hosts for other *Leishmania* spp. [42].

In Europe, canine leishmaniasis (CanL) is endemic in many Mediterranean countries, such as Spain, Italy, Greece and Portugal. In Italy, prevalence varies widely between regions, with the coastal areas of the South reporting the highest rates. Recent studies have shown a northward expansion of the disease, linked to factors such as climate change and the increased mobility of domestic dogs, leading to the emergence of outbreaks in previously non-endemic areas [43,44]. Climate change, increased international travel and the trade of pets have contributed to the spread of the disease to non-endemic areas, as evidenced by cases, for example, in the United Kingdom, Germany, the U.S. or Canada, or in other areas where it has never been recorded before [45,46,47,48].

Beyond the Mediterranean, CanL is also a significant problem in other parts of the world. For instance, in Brazil, CanL is widespread in many regions and poses one of the main challenges to controlling human disease, with control programmes often including drastic measures such as the euthanasia of infected dogs to reduce transmission [49] that still do not solve the issue [5,50].

Leishmaniasis does not spare wild animals, which are affected in large numbers and in various regions of the world. In southern Europe, North Africa and Asia, carnivores such as jackals, wolves and foxes, as well as small mammals such as rodents and bats, have been found to be infected with different Leishmania species. Also in Latin America, some synanthropic rodent species, such as the house mouse or black rat, and marsupials, such as opossums, show high infection rates, suggesting their significant role as reservoirs [51]. The wide geographic distribution and variety of infected species further underline the need for and importance of surveillance of wild animals in order to more accurately understand and manage the zoonotic transmission of the disease [52].

## 3. Leishmaniasis Moves on the Wings of Sand Flies, but What Moves the Epidemiology of Vectors?

In leishmaniasis, we must consider the close interdependence between the vector, the host, the parasite, and the variables capable of altering and reshuffling this relationship: among these, climate stands out. It has been demonstrated that the recent climate crisis, which sees an incessant increase in global temperature for longer and longer periods of time throughout the year, plays an essential role in modifying the epidemiological framework of diseases by altering the life cycle and geographical distribution of vectors [53,54,55]. Changes in environmental conditions indicate that vector-borne diseases are essentially dynamic systems in which their distribution, behaviour, and reproduction exhibit a relevant tendency to adapt. While the spread of leishmaniasis has historically been favoured by tropical and subtropical climates, in recent years, its epidemiology has changed significantly. Sand flies, the vectors of leishmaniasis, are expanding their presence around the world due to climate and other human-made changes that allow them to thrive and reproduce, reaching even historically temperate regions where they were previously sporadic or absent [34]. Other factors besides the climate crisis also play a role. In Latin America conditions have become even more favourable for vectors due to deforestation and movements from rural areas of Brazil to northeastern urban centres [23,43]. In addition, determining factors include urbanisation, globalisation and migration, lack of vector control, travel and international trade. Further factors to consider are population growth and poverty [32,56,57].

Looking more closely at each of these variables, we can say that the survival of vectors and their ability to replicate depend on their interactions with the environment, as well as human and animal activities. Deforestation, closely linked to population growth, has facilitated human-vector contact. International trade and travel connect people but inevitably also vectors and pathogens. The growth of urban areas exacerbates this effect, raising to power the opportunities for contact between people and between people and vectors. For example, in certain Brazilian cities, unplanned expansion has led people to settle in peripheral areas where phlebotomine sandflies are prevalent [58]. A similar situation is observed in Colombia, Venezuela and parts of Southwest Asia [59]. Finally, urbanisation, at least at an early stage, is not synonymous with escaping poverty, which is unfortunately associated with limited access to both treatment and public health tools to prevent exposure to vectors [60].

## 4. A One Health Approach to Surveillance, Prevention and Control

### 4.1. The Importance of Health Promotion and Health Literacy in a One Health Framework

Leishmaniasis has only recently gained increased global attention following the introduction of the Sustainable Development Goals in 2015. In this regard, the World Health Organization (WHO) is committed to the control, prevention, elimination and eradication of NTDs for the period 2021–2030 through the Road Map for NTDs, which aims to reduce the number of people requiring treatment for these diseases by 90% through a plan of integrated approaches and multisectoral coordination [61]. In particular, the roadmap target for the elimination of VL as a public health problem is defined as a mortality rate of less than 1% due to primary VL; for CL, the focus is on reducing morbidity and preventing stigmatisation [62].

Surveillance, prevention and control of any pathology, especially in the case of leishmaniasis, which, as a neglected disease, has historically received poor global attention, can never be fully effective without health promotion strategies capable of increasing health literacy [63,64,65,66], awareness and, consequently, individual empowerment. Policymakers should therefore promote the dissemination of key messages related to actions for surveillance, prevention and control through the implementation of appropriate health policies, including awareness campaigns, ensuring adequate funding, allocation of health resources where needed and organisation of services. In this way, citizens, as active participants, can acquire information and subsequently adopt preventive practices.

In addition to policymakers and the population, to ensure the widespread dissemination of information on how to defend against leishmaniasis, it is essential to involve two key figures: the physician and the veterinarian. Continuous updates should be provided to these health professionals, who are the primary and main figures with whom citizens frequently interface regarding health for themselves and their pets. Their full involvement is also crucial for effective communication tailored to the epidemiology, needs, and specific services of the population and the reference territory.

In this regard, the One Health perspective plays a fundamental role. One Health is “an integrated, unifying approach that aims to sustainably balance and optimise the health of people, animals and ecosystems” that recognises the connection between human, animal and environmental health and promotes collaboration across different disciplines and sectors to more effectively address health threats [67]. To ensure the widespread dissemination of information on how to defend against leishmaniasis, adopting this integrated approach is fundamental. Communication and implementation of preventive and control strategies should therefore be proactively planned and organised at every level: in general medicine offices, travel medicine clinics, public and private veterinary practices, as well as in schools, shelters, grooming centres, rural and urban areas, including specific locations such as parks where both humans and animals are more likely to come into contact with sandflies.

While in many high-income countries, except in certain cases like Portugal [68], public awareness of the growing threat of leishmaniasis still needs to be strengthened, knowledge varies significantly in regions that have long been affected by the disease, particularly in endemic areas of LMICs.

Studies conducted, for example, in Pakistan and Brazil have revealed that the population has limited knowledge of the disease, making any intervention difficult. It is essential to improve public awareness and promote educational interventions to support adequate health practices [69,70]. National health authorities, in collaboration with international organisations such as WHO and the World Organisation for Animal Health (WOAH), should focus on raising awareness of leishmaniasis among the general public and health and veterinary professionals. Clear communication about prevention measures, symptoms, and treatment options is needed to improve community involvement and adherence to public health recommendations. These goals could be achieved through the implementation of up-to-date and useful digital platforms for disseminating information that is understandable to different types of audiences, as well as through social or more classical media. Education and continuing education of health professionals in the human, veterinary, and environmental sectors are critical to improving disease recognition and management.

### 4.2. Practical Measures for Surveillance

The diagnosis of leishmaniasis, both in humans and dogs, is not always straightforward due to the lack of diagnostic tests or insufficient training of healthcare and veterinary personnel, especially in parts of the world where the disease has only recently emerged, in previously non-endemic areas, or where it is still not well known [14,71,72]. Currently, it is estimated that only between 25% and 45% of all new human VL cases are reported to the WHO, while for CL, the number of reported cases ranges from about one-third to one-fifth of the actual cases [1]. There is also a significant underreporting of CanL cases [24,73].

Mandatory reporting practices for all human cases, which are currently absent in many endemic countries, along with passive and active surveillance in dogs, and entomological and environmental monitoring, are critical components in creating a solid integrated and intersectoral One Health surveillance system [74]. These mechanisms are essential for guiding public and veterinary health actions, as the spread of this disease is strongly influenced by the interaction of environmental, human, and animal factors. These surveillance systems must be implemented not only at the local level but also in a coordinated manner across large endemic areas and structured nationally among health authorities. However, there is currently no systematic flow of information that integrates data on humans, animals, vectors, and the environment.

In the Mediterranean basin, where CanL is endemic, an increase in climatic conditions favourable to the transmission of the disease has been observed [75]. By enhancing human, veterinary, and environmental surveillance, as well as improving intersectoral collaboration, the Mediterranean region will be better equipped to manage leishmaniasis. In this context, we will use the Mediterranean basin as an example to explore the topic of integrated surveillance.

#### 4.2.1. Human and Veterinary Surveillance Activities

Inconsistent reporting is a significant challenge. While VL is subject to mandatory reporting in most endemic European countries, exceptions like France and Serbia highlight gaps in the system [76]. Current human surveillance systems are inadequate, leading to global underreporting [1]. Ministries of Health, in collaboration with WHO and the European Centre for Disease Prevention and Control (ECDC), must strengthen frameworks to ensure accurate and timely case reporting. To improve diagnosis and reporting, targeted educational campaigns for healthcare professionals are essential. Training should focus on improving the identification and treatment of leishmaniasis, particularly in areas where the disease is emerging due to environmental or human movement factors. Effective human surveillance requires collaboration with veterinary and environmental health sectors. Establishing permanent communication channels and information-sharing platforms between these sectors will enhance coordination, leading to better public health outcomes. It is therefore important that communication about this disease is accessible to anyone regardless of their social background, and this should clearly be the responsibility of institutions such as the WHO, the National Ministries of Health or veterinary and medical health authorities.

Unlike human leishmaniasis, cases in animals are subject to less rigorous reporting in endemic territories [77]. For this reason, it is essential to organise an effective and mandatory reporting system for animal leishmaniasis cases in all endemic countries of the Mediterranean basin. To be effective, the surveillance network should operate for all dogs, both owned and stray, through integrated zooprophylactic control programmes. The spatial distribution of *L. infantum* infections in dogs is closely correlated with the presence of the disease in humans; this further highlights the need to carefully monitor the canine population both to prevent animal and human outbreaks [24]. The surveillance system should be extended beyond owned animals managed by private clinics or strays managed by local health authorities.

Free or subsidised testing could be offered to breeders and pet owners through partnerships with veterinarians and shelters, ensuring comprehensive data collection on infections in domestic dogs. This approach would help reduce the significant under-reporting of suspected CanL cases in countries such as Greece, where the private veterinary sector handles the majority of diagnoses. One of the main challenges is the limited involvement of the private sector in public health surveillance, which reduces the response capacity of the surveillance system and hinders intersectoral action [78]. Strengthening legal frameworks and ensuring compliance of private veterinary clinics are essential to overcoming this barrier.

#### 4.2.2. Entomological and Environmental Surveillance Activities

Currently, many countries lack a structured and consistent entomological surveillance system. Regular and systematic surveillance of sand fly populations is essential to monitor vector density, geographical distribution and infection rates. These data can provide warning signals for potential epidemics, particularly when climate change creates more favourable conditions for vector-borne diseases such as leishmaniasis.

In particular, a promising approach in entomological surveillance for zoonotic leishmaniasis is xenomonitoring, which involves monitoring insect vector populations for the presence of pathogens within them. In this context, the detection of the parasite in sandflies indicates active circulation between the vector and animal reservoirs, allowing for early intervention before the pathogen spreads to humans. Moreover, xenomonitoring reduces the need for sampling from humans and other reservoir hosts, reducing the costs and logistical challenges associated with conventional surveillance methods.

Systematic surveillance activities incorporating climatic or environmental indicators are currently lacking. The absence of organised entomological surveillance at European level results in significant gaps in knowledge of the distribution, composition and dynamics of vector species [79]. Furthermore, surveillance should encompass factors influencing vector movement, such as climate, to support the development of predictive models for sand fly spread. In this regard, some efforts are already underway, such as the CLIMOS project co-funded by the European Union [80], whose aim is to develop an early warning detection system for sand fly-borne diseases present in Europe and neighbouring countries by monitoring the climatic and environmental factors that influence these vectors. A climate suitability indicator has been proposed to support the surveillance of *L. infantum* in Europe, offering a predictive model to identify areas at risk of transmission [75], and a systematic review of climate-related health indicators has emphasised the importance of including these factors in surveillance systems to address the impact of climate variations on public health [81]. The use of climate suitability indicators to model *Leishmania* transmission will, for sure, offer insights to improve disease surveillance and prevention [82].

#### 4.2.3. Strengthening Leishmaniasis Surveillance Through One Health and Innovation

By improving human, veterinary, and environmental surveillance and promoting intersectoral collaboration, the Mediterranean region will be better positioned to manage the spread of leishmaniasis, in line with a One Health approach. Regular communication and collaboration between public health professionals, veterinarians, entomologists, policymakers and communities are critical to ensure a rapid and coordinated response when increased vector populations or environmental risk factors are detected [83]. Limited financial resources and the lack of permanent mechanisms for intersectoral coordination and communication were identified as key obstacles to the development of an effective surveillance system [79].

An important contribution to addressing this issue could come from the use of artificial intelligence (AI). The ongoing digital revolution offers new opportunities to enhance epidemiological surveillance and monitoring, enabling real-time and systematic tracking of diseases. AI can facilitate the analysis of large volumes of data, identify complex patterns, and predict outbreaks based on environmental and anthropological factors. The WHO is also working to harness the potential of AI as an innovative and equitable tool to improve monitoring capabilities and strengthen the global response to health threats. Therefore, the integration and application of AI could play a crucial role in bolstering surveillance systems and tackling the challenges posed by emerging and re-emerging diseases, including leishmaniasis [84].

### 4.3. Practical Measures for Prevention and Control

In line with the One Health approach, prevention and control policies for leishmaniasis must adopt multidisciplinary interventions. This includes vector management, vaccination, early diagnosis, and treatment of both human and animal cases. In this context, we will use the Mediterranean basin as an example to explore the topic of integrated prevention and control.

#### 4.3.1. Human and Veterinary Prevention and Control Activities

Prevention measures should focus on reducing and possibly avoiding contact between humans, dogs, and sandflies. In some areas of the world, such as Brazil, where VL is endemic, the culling of dogs is a method used to try to control infection. However, there is evidence that euthanasia is not sufficient to control the disease, mainly due to the high infection rate of canine infection and the infectiousness of some infected dogs, the limitations of diagnostic tests in identifying infected dogs and delays between diagnosis and culling [85], as well as being an ethically questionable practice [86]; therefore, it is a practice we do not endorse. An excellent strategy to reduce contact between sandflies and humans and dogs is demonstrated to be the use of insecticide-repellent substances [87]. For instance, deltamethrin collars have been shown to protect dogs from up to 96% of sandflies, specifically against *Phlebotomus perniciosus* [50,88]. This is also important for vaccinated dogs, as they can still transmit the parasite to sandflies despite the vaccination. The use of insecticide-repellent substances on exposed skin is also important for humans [89,90], while another simple but useful means of defence is the use of mosquito nets [91], although it is essential to use nets with sufficiently small meshes to prevent the passage of sandflies, which can easily pass through the larger meshes typical of mosquito nets. Controlling leishmaniasis is particularly complex due to the variety of reservoir hosts involved. Regarding *L. infantum*, controlling reservoir hosts other than dogs, such as rodents, is very challenging. However, dogs can be protected against sand fly bites using long-lasting insecticide-treated collars or spot-on treatments based on pyrethroids, making this an ideal situation for the Mediterranean basin. In contrast, rodents and other wild animals cannot be directly controlled through practical methods like applying repellents. Therefore, it is more effective to focus on reducing the risk of human exposure by managing the environment around homes. This can be achieved by implementing measures to reduce the rodent population in residential areas and improving waste management.

Unquestionably, vaccines are key players in preventive medicine, although they represent a significant challenge in terms of cost and resources [10,92]. For humans, finding an effective vaccine against leishmaniasis has encountered numerous obstacles, including vaccines that have not been able to generate a sufficiently long-lasting cellular immune response, or others that, while live attenuated and capable of inducing long-term protection, present logistical and safety risks, particularly in rural and conflict areas where vaccine storage and distribution are problematic [93].Vaccinating dogs does not prevent them from becoming infected, but it does reduce the likelihood of developing the disease, making the vaccine an important integrated prevention approach. Currently, only one vaccine is available for dogs in Europe, characterised by a good efficacy profile [45]. Sometimes, we might say often, the parasite arrives before the vaccine, making it too late to vaccinate a dog, which is important if the likelihood of the disease occurring is to be reduced. For control actions in this regard, screening programs aimed at the early diagnosis of potential cases should be organised and, where already present, expanded [94].

The approach based on these practices, although time and resource consuming, reduces the risk of transmission, thereby decreasing the disease burden, stigma, disability, and often significant suffering for both humans and animals. In this respect, CanL represents a complex disease that requires a multifactorial therapeutic approach to manage clinical signs, reduce parasite load and avoid relapses. Treatment is mainly based on drugs such as allopurinol, antimonials like meglumine antimoniate, and miltefosine. Alternatively, for dogs with xanthinuria due to side effects of allopurinol, dietary nucleotides with active hexose correlated compound (AHCC) can be considered as an alternative, and domperidone may be used as monotherapy for mild disease [95]. However, the availability of these essential treatments is severely compromised, even in Europe. Many countries, including Austria, France, Germany and Italy, are facing serious difficulties in the supply of veterinary anti-protozoal leishmaniasis drugs, and this deficit has prompted pet owners to increasingly turn to illegal online purchasing channels, exposing dogs to the risk of inadequate or unsafe treatments [96]. For humans, timely treatment of leishmaniasis cases is crucial to prevent severe complications and reduce mortality. In particular, human leishmaniasis’ treatments have seen significant advances in recent years, with the introduction and optimisation of various antileishmanial drugs [97]. The choice of drug depends on the form of the disease, the species of *Leishmania* involved, and the patient’s characteristics. Science closely monitors the effects of these drugs on humans and records the information: among the most notable drugs are pentavalent antimonials, such as meglumine antimoniate, which have long been the mainstay for treating VL, but their use has decreased over the years due to high toxicity and the emergence of resistant parasite strains [98]; liposomal amphotericin B has proven highly effective, significantly reducing treatment duration and side effects [84]; miltefosine, the first oral antileishmanial drug, continues to be a cornerstone of treatment, but concerns about resistance have also emerged [98]. Continued funding for research to develop more effective therapies is necessary. The availability of current drugs is not equally guaranteed worldwide: this depends on the production capacity of pharmaceutical companies, the approval by regulatory agencies in each country, and the financial availability for purchase. For example, the COVID-19 pandemic led to a severe global shortage of liposomal amphotericin B, which was also used to treat mucormycosis, an infection whose incidence increased in India during the pandemic [99]. Overall, access to these drugs is a critical issue, especially in LMICs, where limited availability and access to effective treatments represent a significant challenge for disease control [100], and it is an important issue for all. Economic and logistical difficulties related to drug procurement often hinder optimal management of leishmaniasis, highlighting the need to secure resources and funding, including involvement from private foundations, UN agencies and the WHO [62].

#### 4.3.2. Entomological and Environmental Prevention and Control Activities

Vector management requires systematic and integrated organisation, as outlined by the WHO framework for sandflies’ control in endemic areas, which was instrumental in eliminating VL as a public health problem in Bangladesh, the first country in the world to achieve this [101]. In this context, integrated vector management proves to be a sustainable practice, especially in urbanising areas. This approach includes educational campaigns, integration of vector control methods, intersectoral collaboration, and development of local disease control capacity. Among the recommended measures, a good suggestion from the framework is to monitor vector resistance to insecticides [62]. Educational interventions are particularly effective because they not only increase knowledge of disease symptoms and transmission, but also improve people’s behaviour in controlling vector breeding sites [102]. Community empowerment is critical to ensure the long-term sustainability of interventions, particularly through family involvement in control activities and intersectoral participation. A historical example of successful vector control was the insecticide-spraying campaigns of the 1950s in India, originally aimed at malaria but significantly reducing VL as well [103]. Clearly, this is also applicable for countries in the Mediterranean basin. Integrating multiple control methods allows for the optimisation of costs and intervention efficacy. Control measures include insecticide spraying, the use of treated bed nets, and environmental management. Recent innovations include insecticide-containing wall paints and larvicides, as well as insecticide-repellent combinations for CanL [62]. Establishing an entomological surveillance network, training qualified personnel, and developing regional insecticide reserves are essential for the long-term management of vectors and to prevent leishmaniasis outbreaks in various parts of the world [104].

## 5. Conclusions

In an era of profound global changes, including global warming and climate emergency, large migrations, globalisation, the spread of unsustainable agricultural practices, deforestation and urbanisation, the landscape of infectious and parasitic diseases is changing, with significant repercussions on human health and animals, which are strongly interconnected.

Although leishmaniasis was once confined to poorer and rural regions, it has been in cities and previously unknown places across the world, making it a global threat. NTDs, including leishmaniasis, have long been overlooked in global health policies.

**Figure 4:**
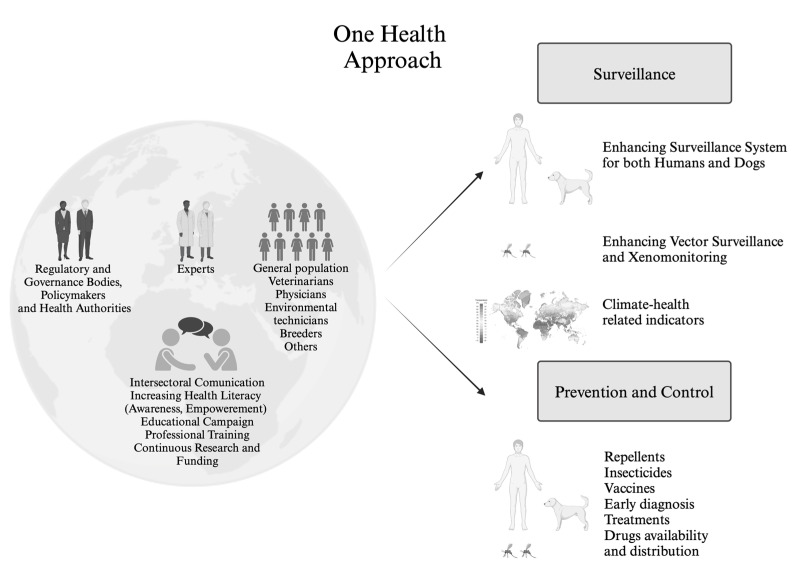
**Leishmaniasis: a One Health approach.** Created in BioRender. Cosma, C. (2024). https://biorender.com/l38m882.

It is essential to adopt an integrated One Health approach (Figure 4) that involves the collection and analysis of epidemiological data from different sources: human, animal, vector as well as climate and their sharing. Integrated prevention strategies, investments in research to develop appropriate technologies and therapies, and appropriate planning are therefore necessary to ensure that these solutions are accessible to all populations. To achieve this goal, it is essential to promote collaboration from a One Health perspective, bringing together public health experts, veterinarians, entomologists, environmental scientists and, last but not least, policymakers. Human, animal and environmental health are interdependent, and collective well-being depends on our ability to maintain this balance.
