# Correction: Cosma et al. Leishmaniasis in Humans and Animals: A One Health Approach for Surveillance, Prevention and Control in a Changing World. *Trop. Med. Infect. Dis.* 2024, *9*, 258

**DOI:** 10.3390/tropicalmed10030074

**Published:** 2025-03-12

**Authors:** Claudia Cosma, Carla Maia, Nushrat Khan, Maria Infantino, Marco Del Riccio

**Affiliations:** 1 Department of Health Sciences, University of Florence, 50134 Florence, Italy; 2 Global Health and Tropical Medicine (GHTM), Associate Laboratory in Translation and Innovation Towards Global Health (LA-REAL), Instituto de Higiene e Medicina Tropical (IHMT), Universidade NOVA de Lisboa (UNL), Rua da Junqueira 100, 1349-008 Lisboa, Portugal; 3 Department of Primary Care and Public Health, School of Public Health, Faculty of Medicine, Imperial College, 90 Wood Ln, London W12 0BZ, UK; 4 Immunology and Allergology Laboratory Unit, S. Giovanni di Dio Hospital, Azienda USL-Toscana Centro, 50012 Florence, Italy

**Error in Figure**

In the original publication [1], there was a mistake in Figure 1 as published. The description of Figure 1 is correct, but two arrows between phases 4 and 5 and between phases 5 and 6 are reversed. In the correct paper, the proper Figure 1 is uploaded.

The corrected Figure 1 appears below. The authors state that the scientific conclusions are unaffected. This correction was approved by the Academic Editor. The original publication has also been updated.

## Figures and Tables

**Figure 1:**
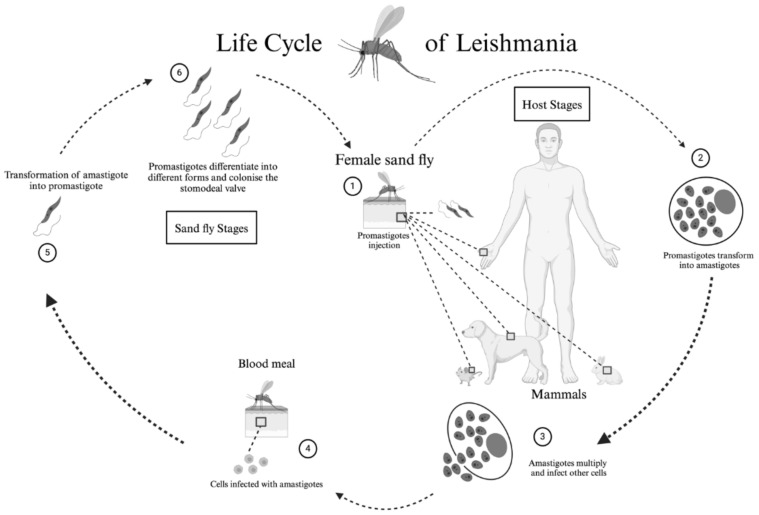
**Life cycle of the *Leishmania* protozoan parasite.** (**1**) The cycle begins when the sand fly inoculates metacyclic (infective) promastigotes into the vertebrate host during a blood meal, along with the fly’s saliva, midgut microbiota, and extracellular vesicles of the parasite. (**2**) The promastigotes are phagocytosed by macrophages and other mononuclear cells, transforming into amastigotes. (**3**) The amastigotes divide and infect other cells. (**4**) The sand fly, during a subsequent blood meal, ingests the infected cells. (**5**) In the midgut of the sand fly, the amastigotes transform into promastigotes. (**6**) The promastigotes differentiate into metacyclic forms and colonise the stomodeal valve. Created in BioRender. Cosma, C. (2024). https://biorender.com/l08n361.
